# A Case Study of Bertolotti’s Syndrome in an Adolescent Patient

**DOI:** 10.7759/cureus.79576

**Published:** 2025-02-24

**Authors:** Lauren N Hoffpauir, Rob Olexo, Hiliary Hamric

**Affiliations:** 1 Research, West Virginia School of Osteopathic Medicine, Lewisburg, USA; 2 Family Medicine, West Virginia School of Osteopathic Medicine, Lewisburg, USA; 3 Pediatrics, West Virginia School of Osteopathic Medicine, Lewisburg, USA

**Keywords:** bertolotti's syndrome, lumbosacral transitional vertebra (lstv), mechanical back pain, pediatric imaging, pediatric spine

## Abstract

Bertolotti’s syndrome is a condition characterized by mechanical low back pain due to a congenital transitional lumbosacral segment of L5-S1. Diagnosis can be challenging for physicians because it requires the presence of both a transitional vertebrae and pain that is directly attributed to this genetic anomaly. This case report describes an adolescent case of Bertolotti’s syndrome seen in a 17-year-old male who presents with several months of low back pain associated with squatting and bending over. His pain was ultimately diagnosed through lumbar radiographs, which demonstrated a transitional L5 vertebrae. Treatment of Bertolotti’s syndrome is dependent on the severity of the symptoms, with options starting conservatively through the use of non-steroidal anti-inflammatory drugs (NSAIDs) and lifestyle modifications to invasive approaches such as nerve ablation or surgical spinal fusion. Bertolotti’s syndrome is rarely diagnosed in children, as most cases of pain begin in early adulthood. However, early recognition in young patients allows for more precisely guided treatments and optimal pain management.

## Introduction

Bertolotti’s syndrome is a condition where back pain is caused by a congenital transitional lumbosacral segment [1]. The first diagnosis of Bertotti’s syndrome was made in 1917 by Mario Bertolotti, who discovered “spatula” shaped transverse processes of L5 were causing pain in patients [2]. Although transitional vertebrae are common radiographically, a diagnosis of Bertolotti’s syndrome can only be made when both pains caused by the spinal anomaly, and a transitional segment are present [1]. This makes a definitive diagnosis difficult. Reports cite that between 4-8% of transitional vertebrae are the confirmed source of back pain and are therefore deemed a true case of Bertolotti’s syndrome [3]. Presentation of the syndrome includes pain in the sacroiliac joints that may or may not radiate into the groin and hip, and has been associated with radiculopathy [1].

## Case presentation

**Figure 1:**
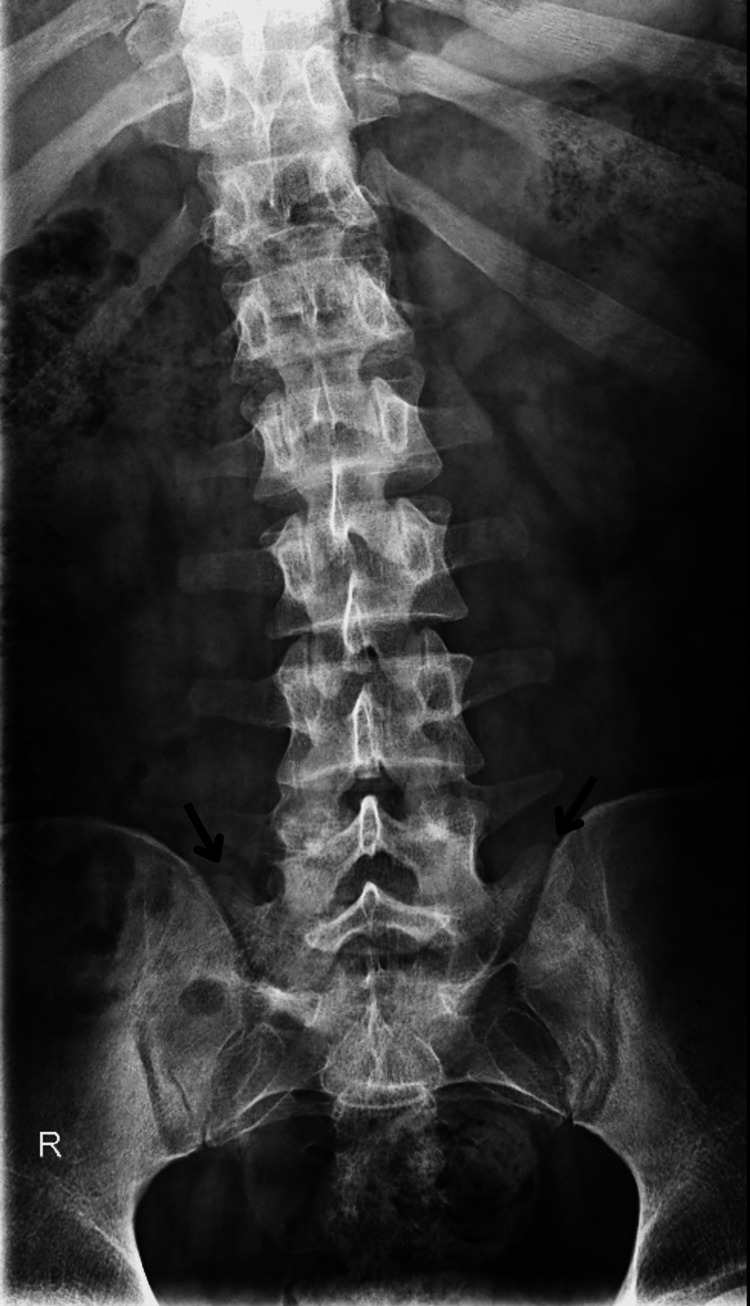
Anteroposterior lumbar spine radiograph demonstrating bilateral transitional transverse processes of the fifth lumbar vertebrae (indicated with black arrows), confirming Bertolotti's Syndrome.

**Figure 2:**
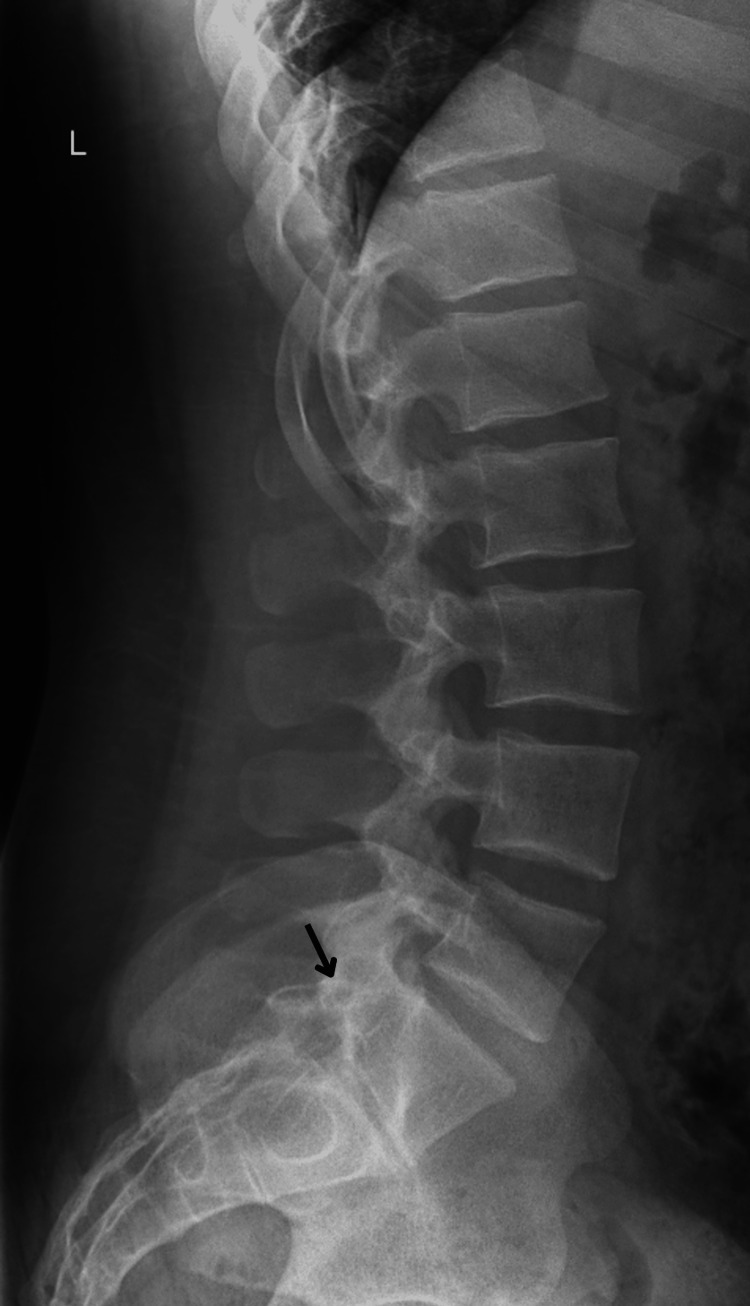
Lateral lumbar spine radiograph with transitional transverse process of the fifth lumbar vertebrae (indicated with black arrow).

A 17-year-old Caucasian male presents to his family medicine doctor’s office for an annual well-child visit with no complaints. The patient’s body mass index is 35 kg/m2. During the examination, the patient is asked about attending high school and his football season. The patient states that he was no longer able to play football due to severe lower back pain while squatting and bending at the waist. He then states that although the pain has been present for a couple of years, recently it has caused him to stop midway through practice and he had difficulty climbing the bleachers to rest. His neurological exam is negative and there are no areas of point tenderness over the spine. The patient states that the pain does not radiate to the groin or cause radiculopathy. He was sent for lumbar spine radiographs (shown below in Figure 1 and Figure 2) and found to have an extended transverse process on the left L5 with arthritic changes in the area. Pain clinical correlates to this patient’s transitional L5 vertebrae during prolonged periods of squatting and forward bending, indicating the presence of Bertolotti’s syndrome, which was confirmed in this patient through radiological imaging.

Treatment for this patient was discussed with both the patient and his parents. After treatment options of over-the-counter pain relief, steroid injections, and nerve ablation were discussed, it was decided that treatment would begin conservatively with non-steroidal anti-inflammatory drug (NSAID) pain reliever use during strenuous activity or episodes of lower back pain. The patient was also advised to limit exacerbating exercises or body movements, such as football, squatting with weights or sustained forward bending, as this was likely to prevent recurrence of the symptoms. The patient presents to the office after several months of cessation of rough contact sports, reporting a complete recovery of low back pain.

## Discussion

Bertolotti’s syndrome is both a clinical and diagnostic diagnosis, where low back pain must correspond to an observable spinal anomaly. Although it is congenital, it is rarely found in pediatric or adolescent patients because the pain is not significant until the patient is age 30 or 40 [1]. Additionally, variations in the anatomy of L5 are two times as common in male patients when compared to females [3]. Bertolotti’s syndrome can be categorized into four types after a diagnosis is confirmed. Type 1 is caused by the extension of the TP (transverse process) of L5, leaving a small gap, at least 19 cm wide, between the TP and ala of the pelvis [1]. Pain is elicited by mechanical friction between the two bones with certain movements of the spine [2]. Type 2 is classified as having direct bone contact between the L5 TP and the ileum of the pelvis, resulting in a pseudoarticulation [2]. This type results in pain that radiates into the deep hip area, due to the direct contact between bones. Type 3 is composed of a fusion of the L5 TP and the pelvis, termed “sacralization” or “lumbarization”, leaving no space for a pseudoarticualtion [1]. Type 2 and 3 can be differentiated by anesthetic and steroid injection into the pseudo-joint space that will either alleviate the pain (type 2) or have no effect (type 3 fusion) [2]. Type 4, or mixed, results from two extending transverse processes, one of which is unilaterally fused [2]. Patients may present with pain on the fused TP and treatment by fusing the opposite side may resolve the pain [2]. Further complications of Bertolotti’s syndrome include disc herniation and degeneration of the L5-S1 disc [3].

Treatment of Bertolotti’s depends on the amount of pain caused and the classification of the transverse process. Type 1 may require conservative treatment with activity modification, or incorporation of anti-inflammatory type pain medications, such as NSAIDs. Types 2 might need further treatment, such as nerve ablation, surgical fusion, or steroid injections directly into the pseudoarticulation [1]. Type 4 will typically require unilateral fusion of the TP that is anatomically unfused at diagnosis [2]. Type 3 is unusual in the fact that the fusion and lack of pseudoarticulation typically do not cause pain, and therefore do not need treatment [2]. The patient in this case was able to modify his level of activity by abstaining from rough contact sports. If pain persists, the patient will be advised to start taking an NSAID medication during episodes of discomfort.

## Conclusions

Bertolotti’s syndrome is likely underdiagnosed and commonly incidentally found on radiographs, especially in young children or teenagers. The four types of Bertolotti’s syndrome have a diverse range of presentations and treatment needs to be determined on a case-by-case basis. Although most cases will present in mid-adulthood, young kids with positional back pain should be sent for simple anteroposterior and lateral plain film radiographs to assess for potential spinal anomalies. Not only can small lifestyle modifications prove beneficial for adolescent children with chronic back pain, but early diagnosis can also assist with treatment planning for the child as they progress into adulthood and spinal arthritic changes begin. More advanced types of Bertolotti’s syndrome will likely need more invasive forms of treatment, but are manageable with injections, ablations and anti-inflammatories.
